# Extrapyramidal signs in normal pressure hydrocephalus: an objective assessment

**DOI:** 10.1186/1743-8454-4-7

**Published:** 2007-08-13

**Authors:** Allen S Mandir, Jennifer Hilfiker, George Thomas, Robert E Minahan, Thomas O Crawford, Michael A Williams, Daniele Rigamonti

**Affiliations:** 1 Johns Hopkins University, Department of Neurology and Neurosurgery, Baltimore, MD 21287, USA; 2 Georgetown University Hospital, Department of Neurology, 3800 Reservoir Road – PHC-7, Washington, DC 20007, USA; 3 LifeBridge Health Brain & Spine Institute, Adult Hydrocephalus Center, Sinai Hospital of Baltimore, Baltimore, MD, 21215, USA

## Abstract

**Background:**

Beyond the classic Normal Pressure Hydrocephalus (NPH) triad of gait disturbance, incontinence, and dementia are characteristic signs of motor dysfunction in NPH patients. We used highly sensitive and objective methods to characterize upper limb extrapyramidal signs in a series of NPH subjects compared with controls. Concentrated evaluation of these profound, yet underappreciated movement disorders of NPH before and after techniques of therapeutic intervention may lead to improved diagnosis, insight into pathophysiology, and targeted treatment.

**Methods:**

Twenty-two (22) consecutive NPH patients and 17 controls performed an upper limb motor task battery where highly sensitive and objective measures of akinesia/bradykinesia, tone, and tremor were conducted. NPH subjects performed this test battery before and more than 36 h after continuous CSF drainage via a spinal catheter over 72 h and, in those subjects undergoing permanent ventriculo-peritoneal shunt placement, at least 12 weeks later. Control subjects performed the task battery at the same dates as the NPH subjects. Statistical analyses were applied to group populations of NPH and control subjects and repeated measures for within subject performance.

**Results:**

Twenty (20) NPH subjects remained in the study following CSF drainage as did 14 controls. NPH subjects demonstrated akinesia/bradykinesia (prolonged reaction and movement times) and increased resting tone compared with controls. Furthermore, the NPH group demonstrated increased difficulty with self-initiated tasks compared with stimulus-initiated tasks. Following CSF drainage, some NPH subjects demonstrated reduced movement times with greater improvement in self- versus stimulus-initiated tasks. Group reaction time was unchanged. Resting tremor present in one NPH subject resolved following shunt placement. Tone measures were consistent for all subjects throughout the study.

**Conclusion:**

Clinical motor signs of NPH subjects extend beyond gait deficits and include extrapyramidal manifestations of bradykinesia, akinesia, rigidity, and propensity to perform more poorly when external cues to move are absent. Objective improvement of some but not all of these features was seen following temporary or permanent CSF diversion.

## Background

The classic clinical triad of Normal Pressure Hydrocephalus (NPH) includes gait disturbance, incontinence, and dementia though most patients exhibit less than three of these signs early in the progressive clinical course [1,2]. In addition to classic gait abnormalities, NPH patients exhibit other motor deficits including those affecting the upper limbs. Extrapyramidal motor signs in NPH patients include Parkinsonian and Huntingtonian movements, and these signs may reverse following therapeutic cerebrospinal fluid (CSF) drainage [3,4].

That ventriculo-peritoneal shunting reverses each of the classic signs in some NPH patients suggests a causative role in CSF dynamics, but the mechanisms underlying the neuronal and glial pathophysiology of NPH remain obscure. During the course of NPH, periods of elevated intracranial CSF pressure are hypothesized to result in dysfunction of white and gray matter of the brain (including basal ganglia) [5]. These hypotheses are supported by functional imaging studies as well as post-mortem pathologic analysis of brain parenchyma [2,6-8]. At the time of diagnosis, CSF pressures may be "normal" with accommodating ventricular enlargement over time; alternatively, intermittently elevated pressures may go undetected in NPH unless continuous CSF pressure monitoring is performed [9].

Few investigations of NPH patients have included objective and sensitive analysis of extrapyramidal motor signs, and examination of these signs outside of gait dysfunction are rarer still [3,10-12]. We sought to examine a series of subjects referred for evaluation of possible NPH at baseline, following CSF drainage via spinal catheter and again following placement of a permanent ventriculo-peritoneal shunt using objective, sensitive measures of extrapyramidal motor signs. Using a battery of non-invasive tests, we report our results of upper extremity movement initiation and execution, tone and tremor measures during baseline and following these therapeutic interventions. Control cohorts were examined using the same testing battery but without any intervention. The results of these studies should serve as a basis for insight into the mechanisms of disease, improve clinical recognition and objectively demonstrate features of upper extremity motor performance improvement following potential therapeutic intervention.

## Methods

### Demographics

This study, together with patient recruitment, was approved by the Johns Hopkins University Institutional Review Board. Signed, informed consent was obtained from each subject. Twenty-two (22) consecutive subjects above the age of 50 (average age 74.7; range 51 – 85 years) referred for evaluation of possible NPH were recruited from the Johns Hopkins University Outpatient Center, with no enrollment restrictions based on race or ethnic origin. Requirements for inclusion were radiographic evidence of ventriculomegaly by CT scan or conventional MRI, and clinical signs of NPH that included gait impairment, urinary incontinence, or cognitive impairment. Subjects were excluded if they had undergone prior shunt or intracranial surgery, were unable to undergo lumbar puncture for CSF drainage or had other active neurologic disease (stroke, Parkinson's disease, Alzheimer's disease, CNS infection, blindness, myelopathy or amputation). Controls (n = 17) were partners of eligible NPH subjects (average age 70; range 57 – 79 years) and were required to be free from clinical signs of NPH.

NPH subjects were tested with the entire battery of tasks at 1) baseline, 2) within 36 h following removal of approximately 10 ml CSF/h for 72 h via a temporary spinal catheter and 3) approximately 12 weeks following ventriculo-peritoneal shunt insertion (Medtronic). Control subjects were tested with the entire battery of tasks on the same days of testing as NPH subjects. Two NPH subjects demonstrated signs of infection following CSF drainage via spinal catheter and were excluded from further testing and analysis. One subject withdrew consent to participate in the study after baseline testing such that subsequent data were not collected.

### Volitional motor tasks

Subjects performed ballistic movements using their dominant hand by moving a manipulandum that constrained two-dimensional movement about the wrist. Targets, 10° wide and unbounded (to require precision in holding initial and final target positions), were separated by 50°. Practice trials were performed for this simple task to avoid any potential learning curve barriers. Subjects reacted and moved in response to an auditory "GO" signal (stimulus-initiated tasks) or performed the identical movement task in the absence of a "GO" signal (self-initiated tasks). All subjects were informed ahead of time if they were to perform stimulus- or self-initiated tasks, and in both cases received preparatory auditory cues of "ready" and "set" in the initial stages of the task. In stimulus-initiated tasks, these preparatory cues preceded randomly delivered "GO" signals, and in self-initiated tasks, where no "GO" signal was delivered, a minimum time of 2 s of hold time was required after the "set" cue before initiating movement. Errors were recorded and the task discarded if movement occurred prior to 150 ms more than "GO" signal, more than 1 s after the "GO" signal, or if final target was overshot. An electronic goniometer continuously recorded manipulandum position during the task and was fed into an online recording computerized system (Biopac Systems, CA) along with auditory cues and electromyographic recordings of agonist and antagonist wrist muscles performing the task. Forty-five correctly performed trials were performed by each of the subjects. Reaction time (RT) was measured as time between "GO" signal and first significant change of the agonist electromyographic record (EMG) over baseline (RT/EMG) and between "GO" signal and movement onset (MO) (RT/MO). Electromechanical delay (EMD) was determined as the time between first agonist EMG and movement onset. Movement time (MT) was defined as the time between movement onset (MO) and achieving final target.

### Tone measures

Muscle tone was measured by an apparatus that quantifies tone about the elbow. Subjects rested their dominant forearm in a molded and padded cradle mounted on a metal beam. The cradle rotated on low-friction ball bearings and an electronic goniometer measured angular displacement. A load cell (Biopac Systems, CA) located at the side of the metal beam measured the torque applied by the examiner. The torque plotted against the angle (degrees) represented muscle stiffness, and post-hoc least squares fit analysis was then performed to determine the slope of the contour representing tone about the elbow [13]. The tone apparatus allowed the examiner to test the arm using movements similar to that during a clinical exam, whereby the examiner flexes and extends the patient's elbow while at repose. Data were rejected if there was active resistance or assistance by the patient as in 'gegenhalten', which was detected by aberrations in the slope of the stiffness (stiffness constant *k *= the slope of force over displacement), or active EMG activity in primary agonists or antagonists about the elbow [13]. Three data sets of continuous, repetitive arm flexion and extension motion were obtained from each subject until a reproducible trace was obtained [13].

### Tremor analysis

Tremor was analyzed using triaxial solid-state accelerometers (Biopac Systems, CA) that detect frequency (Hz) and amplitude (root mean square horizontal and vertical displacements) in μm. Triaxial accelerometers were placed on the distal hand while the subjects' hands were at repose for a period of 120 s. Subjects were then asked to hold their arms outstretched, and tremor analysis was repeated during the next 60 s. All data were fed online to a Pentium based computer and analyzed using Fast Fourier Transforms. Mean tremor amplitude and dominant frequency were recorded while at rest and with outstretched arms.

### Statistical methods

Group statistical analyses for parametric measures were compared using ANOVA tests with Fisher's Least Square Difference Post-Hoc analysis when F scores were significant or Kruskal-Wallis comparisons for non-parametric measures of reaction time. Analyses within individuals were compared using Repeated-measures ANOVA with Fisher's Least Square Difference or Friedman's analysis with Mann-Whitney Post-Hoc analysis.

## Results

### Simple reaction time

**Figure 1:**
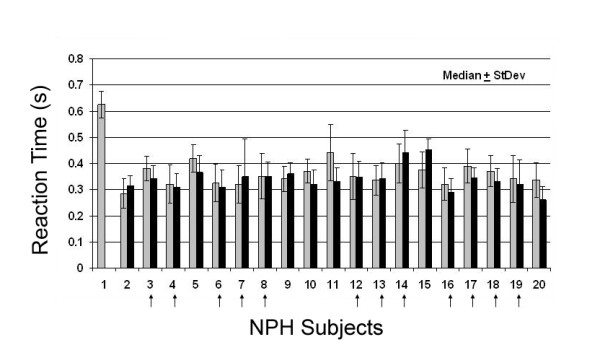
**Reaction time in NPH**. Plot of Reaction times to movement onset (RT/MO) for each NPH subject at baseline (gray bars) and following CSF removal (black bars; n = 45). Data are median values ± standard deviation in s. No significant differences were demonstrated in NPH RTs following CSF drainage via a temporary spinal catheter (nor later by VP shunt). Vertical arrows indicate those NPH subjects who later underwent shunt placement.

As a group, NPH subjects demonstrated modest but significant prolongation of reaction time measured either as RT/EMG (controls: 170.0 ± 39 ms v/s NPH subjects: 233.8 ± 34 ms; median ± SD; *p *< 0.01), or as RT/MO (controls: 299.0 ± 38 ms v/s NPH subjects: 345.8 ± 39 ms; median ± SD; *p *< 0.01) when compared with the control group (Table 1). However, there is overlap between individual controls and NPH subjects in RT measures (range for controls: 245.3 – 361.3 ms; range for NPH: 282.8 – 434.2 ms). Few NPH subjects demonstrated significant reductions in RT whether measured by RT/MO or RT/EMG following CSF drainage and equally few demonstrated prolonged RTs (*p *< 0.05; Friedman's analysis) (Figure 1). As a group, the change in RT/MO was similar to that of RT/EMG, but for a few subjects the improvement in RT/EMG exceeded that for RT/MO. This suggests for these few subjects that pure reaction time decreased as measured from "GO" signal to agonist EMG activity, but electromechanical delay prevented decreased movement initiation latency. This was likely caused either by co-contraction (antagonist muscle activity interfered with movement initiation) or less than optimal muscle organization preventing earlier movement-initiation. Controls did not reveal significant changes in RT measures on repeat visits.

**Table 1:** Overview of grouped data represented as means of medians (RT) or means of means (MT; Tone) ± SEM. (When taken as a group, NPH subjects performed differently from control subjects in each measure; ** *p *< 0.01, * *p *< 0.05).

	RT/EMG (ms)	RT/MO (ms)	Stim MT (ms)	Self MT (ms)	Tone (*k*)
Controls	170.0 ± 39	299.0 ± 38	493 ± 15	513 ± 18	.0012 ± .0003
NPH	233.8 ± 34**	345.8 ± 39**	554 ± 26**	628 ± 39**	.0055 ± .0010*

### Ballistic movement times

**Figure 2:**
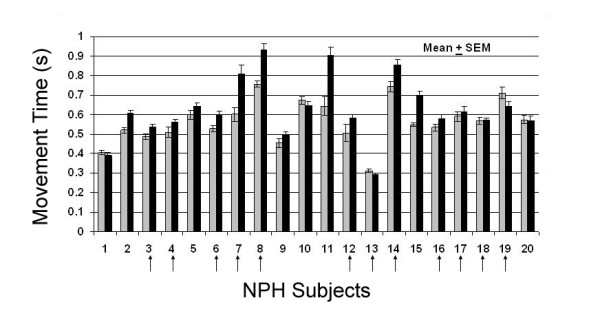
**Movement time in NPH**. Plot of stimulus-initiated (gray bars) and self-initiated (black bars) movement times (MT) of each NPH subject at baseline in s (mean ± SEM; n = 45). A subset of subjects demonstrated statistically prolonged movement times during the self-initiated tasks compared with stimulus-initiated tasks. Vertical arrows indicate those NPH subjects who later underwent shunt placement.

As a group, NPH subjects demonstrated prolonged MT versus controls (Table 1). At baseline, a subset of NPH subjects demonstrated significantly prolonged movement times when required to make self-initiated compared with stimulus-initiated movements to an unbounded target (repeat measures ANOVA; *p *< 0.02; F = 3.824). Mean movement times for stimulus-initiated and self-initiated tasks are plotted for each NPH subject at baseline in Figure 2. Approximately 40% of NPH subjects exhibit this behavior that self-initiated tasks were prolonged compared with stimulus-initiated tasks, whereas less than 15% of control subjects exhibited this behavior. The absolute magnitude of this discrepancy is greatly enhanced in NPH compared with those controls that exhibit minor differences between self- and stimulus-initiated motor behavior.

Those NPH subjects with fastest movement times (least bradykinetic) were least apt to exhibit discrepancies between self- and stimulus-initiated movements. Furthermore, those NPH subjects with fastest movement times were least apt to exhibit wide intertrial movement time variability as reflected by size of error bars (Figure 2).

**Figure 3:**
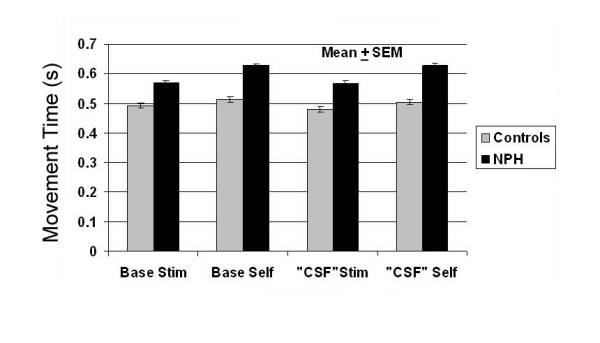
**Combined movement time in control and NPH subjects**. Plot of mean combined movement times (s) of all Control and NPH subjects at baseline and at a time following CSF drainage in NPH subjects with controls retested at the same time (means ± SEM). As a group, NPH subjects were bradykinetic compared with controls and demonstrated greater disparity between self- and stimulus-initiated movement times. As a group, no significant improvement was seen in MTs following CSF drainage in NPH subjects, but repeat measures analysis demonstrated significant improvement in a subgroup.

Following CSF drainage, NPH subjects demonstrated improvement in self-initiated tasks as compared with stimulus-initiated tasks (5 of 19 subjects; repeat measures ANOVA; *p *< 0.05), but this did not hold true for the group analysis comparing NPH subjects before and after continuous CSF drainage (Figure 3). Figure 3 reflects that NPH subjects exhibit overall bradykinesia compared with controls as well as an increased propensity of NPH subjects to perform more poorly on self-initiated tasks compared with stimulus-initiated tasks.

**Figure 4:**
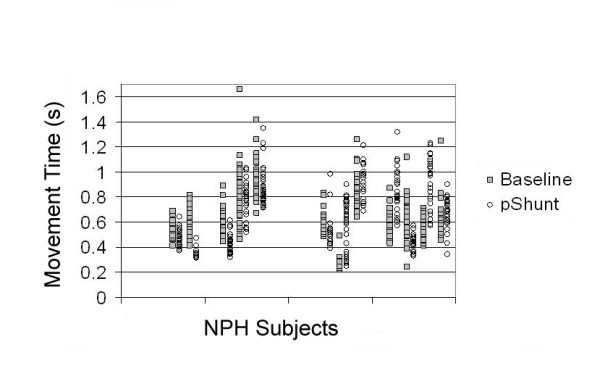
**Self-initiated movement in NPH**. Raw data plots of the 12 NPH patients who received V-P shunt during self-initiated movement time (MT) tasks. The Y axis represents time in s, separate patients are plotted on the X axis with MT from baseline measure (gray squares) and following V-P shunt placement (open circles). Those subjects that demonstrated changes in MTs (whether prolonged or decreased) with CSF drainage, demonstrated similar changes from baseline. (For comparison, average control self-initiated movement times were 513 ± 18 ms).

Based on the clinical response to CSF drainage, some NPH subjects were offered and elected to receive placement of a ventriculoperitoneal shunt (n = 12). Those subjects demonstrating significant differences of movement times from baseline following CSF drainage maintained this difference following shunt placement. Furthermore, selective improvement of self- versus stimulus-initiated movement times persisted with shunt placement (Figure 4). Control subjects did not demonstrate significantly different performances on any of the motor tasks with repeated measures on subsequent visits, compared to baseline (repeat measures ANOVA

### Rigidity in NPH

**Figure 5:**
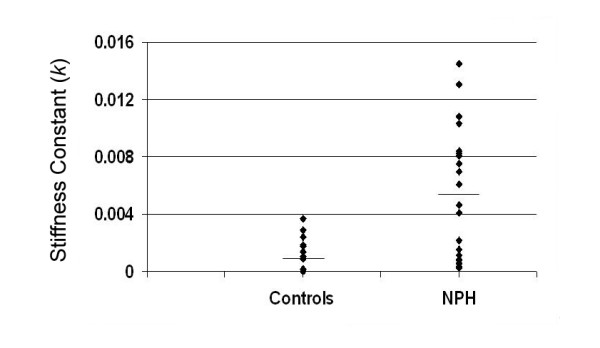
**Tone analysis in NPH versus control subjects**. Plots of the slope of the line of force: angular displacement about the elbow (stiffness constant *k*) for control and NPH subjects at baseline during repose. Solid diamonds represent each individual subject, and horizontal lines denote mean values. As a group, NPH patients displayed significantly increased tone versus controls (*p *< 0.02) with a majority of subjects demonstrating tone outside the range of controls.

Tone measures at baseline demonstrate increased passive tone about the elbow in NPH subjects compared with control cohorts (*p *< 0.02, Table 1, Figure 5). The source of increased resting tone was not spasticity or gegenhalten. Consistency of tone testing within all subjects was supported by consistent hysteresis loops of force versus distance with highly correlative measures between testing on different days for controls (linear regression; *p *< 0.02, R^2 ^= 0.79). Following CSF drainage and following shunt placement, there were also no significant changes in slope (representing rigidity at the elbow) for the NPH subjects. However, there were qualitative differences in tone hysteresis loops following CSF drainage in NPH subjects including greater incidence in EMG activity and sudden tone perturbations in the NPH group not seen in control subjects.

Correlations between increased rigidity and bradykinesia were poor amongst NPH subjects, such that these seem to be expressed independently within subjects. Similarly, improvements in bradykinesia were not reflected in reductions in rigidity following therapeutic interventions.

### Tremor studies

Controls and NPH subjects were largely without detectable tremors either at rest or with sustained arm postures. The exceptions were in two NPH subjects who demonstrated low amplitude, low frequency tremor. Of the two subjects with tremor, following shunt placement, rest tremor was no longer detectable in one subject. Even with the highly sensitive accelerometer, tremor did not appear in the majority of NPH subjects tested.

### Correlation with classic NPH parameters

In a parallel study, these same subjects underwent formal gait analysis (Williams *et al*, unpublished data). Although some subjects improved in both gait and upper extremity measures, this was not uniformly so – such that substantial improvement in gait measures did not always coincide with improvement in ballistic measures. Since none of the subjects improved in measures of tone, improvement in gait did not correlate with this measure. Interestingly, some of those subjects that demonstrated increased upper extremity bradykinesia following CSF drainage also demonstrated worsening bradykinesia in gait testing.

## Discussion

Objective assessment of upper limb movement performance is lacking for NPH subjects. No prior study of objective, quantifiable measures of tone in NPH subjects has been reported. Using our sensitive measures we detected bradykinesia, akinesia and increased tone in NPH subjects compared with controls. By our novel testing paradigms in NPH subjects employing both stimulus- and self-initiated tasks, we also found that some NPH subjects demonstrate the classic Parkinsonian motor characteristic of improved movement execution in the presence of external cues. Following CSF drainage by temporary spinal catheter or permanent shunt placement, bradykinesia seemed most amenable to improvement. In NPH subjects, motor performance in self-initiated tasks showed greater dysfunction at baseline than with stimulus-initiated tasks, and demonstrated more dramatic improvement following CSF drainage.

There are several anecdotal reports of NPH causing Parkinsonian motor behavior [4,14,15]. While it may be argued that these patients may have both NPH and idiopathic Parkinson's disease, reversal of these signs with CSF drainage and the general failure of these patients to improve with levodopa therapy argues against dual diagnoses. Our more detailed and sensitive investigation of arm motor performance demonstrated that a majority of NPH subjects exhibit Parkinsonian signs, with bradykinesia being most common. Other uncommon extrapyramidal presentations in NPH include Huntingtonian signs that may reverse following shunt surgery [16]. These reports and our data suggest that extrapyramidal pathways are often affected in NPH pathophysiology.

Although NPH has been formally described for over 4 decades, the pathophysiology and etiology underlying this disorder remain obscure. Several hypotheses exist for the motor manifestations of the disease. The derangement of anatomical structures from increased ventricular size is thought to exert shearing forces on underlying parenchyma [5]. Those structures immediately adjacent to enlarged ventricles may be subjected to the greatest stress. Since subcortical white matter corresponding to leg motor fibers are most medial, it is suggested that these may be most adversely influenced. However, it is clear by this and other studies that NPH subjects exhibit signs beyond gait problems, including a variety of extrapyramidal signs [4]. In this study, the NPH subjects as a group demonstrated akinesia, bradykinesia and increased tone compared with controls.

White matter influences on extrapyramidal loops, or on connections in premotor processing theoretically explain these Parkinsonian signs. However, there is evidence in NPH post mortem analyses that deep brain nuclei involved in extrapyramidal motor control are also directly affected. Indeed, although some PET studies suggest white matter dysfunction in NPH as inferred from reduced cerebral blood flow, there are conflicting studies throughout the literature to these findings [6,8,17]. At least one recent PET analysis suggested a predominance of basal ganglia dysfunction on PET in NPH subjects [17]. Anatomical studies on post-mortem NPH brains also demonstrate pathologic change in both white matter as well as basal ganglia parenchyma [7]. It is hypothesized that shearing forces may have direct and indirect influences upon brain parenchyma [5]. Shearing stress may compromise tissue function directly, but may also underlie a process of small vessel hyalinization to cause later dysfunction; this may occur especially in those basal ganglia structures most at risk for this process [5,7].

No correlations exist between baseline presentation of tone, akinesia or bradykinesia (whether examining self- or stimulus-initiated movements) in NPH subjects. This implies that different pathophysiological substrates underlie each of these motor signs in NPH, and/or that these signs emerge independently. Of these extrapyramidal signs, not all respond equally to CSF drainage. Why would only some symptoms respond to CSF drainage? It is plausible that some structures revert to normal function with reduction of direct shearing forces, while others (say with ischemia from small vessel hyalinization) are irreversible or require time for tissue recovery or compensatory pathways to develop. In addition, there is always a potential for multiple etiologies to confound the presentation and responsiveness of clinical signs related to NPH, but our selection criteria sought to minimize these factors.

It is an interesting finding that some NPH patients demonstrated the classic Parkinsonian motor characteristic of improved movement execution in the presence of external cues. Others have seen a less dramatic benefit of external cues when studying NPH patients during gait analysis [10,18]. Our study design sought to include direct comparison of self- and stimulus-initiated movements to directly examine the influence of external cues in motor performance. The underlying mechanism of improved motor performance with external cues in Parkinsonism is suggested to arise from dysfunction of the supplementary motor area (SMA). The SMA is credited with motor initiation and planning of movement and is disrupted in Parkinsonism [19]. This cortical structure is dependent upon basal ganglia input via thalamus and in turn has direct influence over other premotor areas as well as the primary motor cortex. Furthermore, the SMA is anatomically situated similar to the leg homunculus representation of the primary motor cortex, readily susceptible in its subcortical projection and receiving fibers to potential shearing forces from ventricular enlargement as seen in NPH. Future correlations of our objective, sensitive neurophysiological measures with pathological and advanced imaging techniques may help correlate specific SMA and surrounding structure dysfunction with increased bradykinesia during self-initiated movements. In addition, this may lead to better predictive value of patient improvement with therapeutic interventions related to CSF flow.

NPH subjects demonstrate increased tone compared with controls. This tone is unrelated to spasticity and independent of movement speed. Our measures of tone are highly sensitive, and even a significant increase in tone compared with controls may be subclinical. From what is known of NPH, one would expect a supraspinal mechanism underlying increased tone. Such mechanisms have been proposed for the increased tone seen in Parkinsonism [20]. There was no correlation between increased tone and the presence of other extrapyramidal features at baseline in NPH subjects. Measures of tone did not improve following CSF drainage. Thus neuronal pathways, other than those directly relieved by CSF drainage may be responsible for rigidity in NPH, or those contributing underlying factors contributing to tone are relatively non-reversible, say by ischemic changes.

## Conclusion

The underlying motor pathophysiology of NPH signs extends beyond gait and bladder dysfunction with prevalence of extrapyramidal signs. These include even complex Parkinsonian signs of reliance upon external cues for improved motor performance. That NPH subjects demonstrated improvement of some signs in short order following CSF drainage suggests reversible pathophysiologic mechanisms that can improve activities of daily living. However, sensitive and objective motor measures in this sample suggest other motor dysfunction is resistant to immediate correction by CSF drainage and may be recalcitrant or require chronic treatment to see improvement.

## Competing interests

Dr. Michael A. Williams and Dr. Daniele Rigamonti have received honoraria in the past from Medtronic, Inc. Dr. George Thomas worked at the Adult Hydrocephalus Center at Johns Hopkins when this study was conducted. His salary was supported by a grant from Medtronic, Inc.

## Authors' contributions

ASM provided study design, oversight of data acquisition, data analysis, and drafting the manuscript. JH tested each subject, performed data collection and first stage data analysis. GT provided subject recruitment, scheduling and evaluation. REM participated in critical review of the manuscript and in evaluation of study design. TOC participated in provided testing environment and critical analysis on data collection and interpretation. MAW contributed in patient evaluation and categorization and critical manuscript review. DR contributed to study design, patient evaluation and critical manuscript review. All authors have read and approved the final manuscript.
